# Categorization of tinnitus listeners with a focus on cochlear synaptopathy

**DOI:** 10.1371/journal.pone.0277023

**Published:** 2022-12-13

**Authors:** Chiara Casolani, James Michael Harte, Bastian Epp

**Affiliations:** 1 Auditory Physics Group, Hearing Systems Section, Department of Health Technology, Technical University of Denmark, Lyngby, Denmark; 2 Interacoustics Research Unit, Lyngby, Denmark; 3 Eriksholm Research Centre, Snekkersten, Denmark; University of Michigan, UNITED STATES

## Abstract

Tinnitus is a complex and not yet fully understood phenomenon. Often the treatments provided are effective only for subgroups of sufferers. We are presently not able to predict benefit with the currently available diagnostic tools and analysis methods. Being able to identify and specifically treat sub-categories of tinnitus would help develop and implement more targeted treatments with higher success rate. In this study we use a clustering analysis based on 17 predictors to cluster an audiologically homogeneous group of normal hearing participants, both with and without tinnitus. The predictors have been chosen to be either tinnitus-specific measures or measures that are thought to be connected to cochlear synaptopathy. Our aim was to identify a subgroup of participants with characteristics consistent with the current hypothesized impact of cochlear synaptopathy. Our results show that this approach can separate the listeners into different clusters. But not in all cases could the tinnitus sufferers be separated from the control group. Another challenge is the use of categorical measures which seem to dominate the importance analysis of the factors. The study showed that data-driven clustering of a homogeneous listener group based on a mixed set of experimental outcome measures is a promising tool for tinnitus sub-typing, with the caveat that sample sizes might need to be sufficiently high, and higher than in the present study, to keep a meaningful sample size after clustering.

## Introduction

Subjective tinnitus, the perception of a phantom sound in the absence of an external stimulus, is a complex phenomenon whose causes and mechanisms are not yet completely understood. Due to a lack of standardization to assess tinnitus and due to multiple definitions of the phenomenon, the real prevalence of tinnitus is largely unknown. Studies report prevalence ranging from few percent to around 30% [1]. The American Tinnitus Association estimates that around 15% of Americans have subjective tinnitus, which, differently from objective tinnitus, is not related to blood flow or muscular-skeletal mechanisms [2]. Despite this, tinnitus research is still very inconclusive, and treatment outcomes are, in many cases, only placebo effects [3].

One of the challenges is that much of the current literature on tinnitus sufferers include listeners with tinnitus and a hearing loss [4], which potentially complicates the interpretation of the results. With respect to the mechanisms, hearing loss can roughly be divided into sensorineural hearing (SNHL) loss and conductive hearing loss (CHL). SNHL is mainly defined as reduced audibility due to damage of the inner ear or the auditory nerve and attributed to aging or induced by noise. CHL is related to problems in the transmission of the sound either in the external ear (e.g cerumen impaction) or in the middle ear (e.g fluid presence). Regardless of the type of hearing loss, tinnitus can also be present. Moreover, hearing loss and tinnitus are also two possible symptoms of Meniere’s disease and acoustic neuroma [5]. In addition, in human studies the otologic background of each subject can be difficult to assess. A characterization of tinnitus in homogeneous subgroups would be helpful to understand the underlying mechanisms behind the issue and proceed to the development of focused treatments [3].

One approach to shed light on the mechanisms underlying tinnitus is to identify biomarkers that are independent of any subjective reporting of the listener. The results of different studies aimed to find biomarkers for tinnitus are inconsistent and often suggest opposing theories to explain the observed phenomena. However, a recurrent hypothesis associates tinnitus with an increased neural firing that could be either more centrally located or in more peripheral locations [6]. Non-invasive electrophysiological and imaging techniques like electroencephalography (EEG) [7], auditory brainstem response (ABR) [8–10], magnetoencephalography (MEG), functional magnetic resonance imaging (fMRI) [11, 12] and positron emission tomography (PET) have been widely used to try to test this hypothesis and localize the source of the tinnitus.

**Fig 1:**
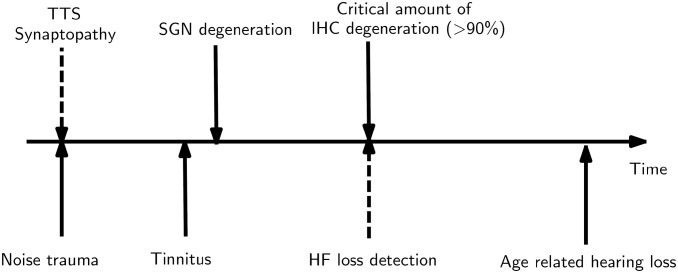
Assumption behind the connection between tinnitus, cochlear synaptopathy and high-frequency hearing loss. The assumption is that a noise trauma triggers a temporal threshold shift, accompanied by cochlear synaptopathy. With time, the thereby reduced input into the brain stem leads to tinnitus and to spiral ganglion neuron (SGN) degeneration. Progressive effects of synaptopathy and SGN degeneration lead to high-frequency hearing loss under the assumption of higher vulnerability of the basal part of the cochlea. The same timeline might apply to age-related cochlear synaptopathy and shortened by noise overexposure.

One potential mechanism for reduced input into the brain is the deafferentation of auditory nerve fibers (cochlear synaptopathy, CS) in the inner ear. This might lead to a compensatory increase in neural gain, which itself could cause tinnitus [13]. Numerical simulations have shown that CS might lead to overrepresentation of specific frequencies which might be one mechanism underlying tinnitus [8]. One of the main assumptions underlying this appraoch is that a noise trauma triggers the subsequent degeneration of spiral ganglion neurons (SGN) (Fig 1). This degeneration will not immediately be visible in the audiometric thresholds, but first be evident after a critical time and after a critical amount of SGN degeneration has been reached [14, 15]. It has also recently been shown in human temporal bones, that synaptopathy highly correlates with age, suggesting it being omnipresent also in normal hearing listeners [16].

A recent study showed that tinnitus sufferers had lower middle ear muscle reflexes (MEMRs) compared to a control group [17]. This suggested that the presence of tinnitus might be related to the functioning of the efferent system which, in turn, might be connected to CS. The use of MEMR as a measure of synaptopathy-related tinnitus is particularly promising both for being fast and for being a physiological objective biomarker. However other studies showed no connection between MEMR and tinnitus [18].

Besides physiological measures, behavioral measures might contribute to identifying subgroups of mechanisms underlying tinnitus.

A combination of assessing tinnitus frequency, tinnitus loudness, psychophysical tuning curves (PTC) and tinnitus tuning curves (TTC) revealed differences in a group of tinnitus listeners [19]. This variability potentially provides information about the tuning properties of the system and thereby the potential mechanism underlying tinnitus. As for synaptopathy, high frequency audiometry (HFA) has been selected as one proxy of the presence of synaptopathy as animal models showed a higher prevalence for synaptopathy at tonotopic places connected to high frequencies [20]. Finally, adaptive categorial loudness scaling (ACALOS) was reported to provide indications about the presence of hyperacusis, that has been suggested to be an important confound to take in account [21].

To develop a screening procedure for tinnitus which allows tinnitus subtyping, the selection of effective indicators of tinnitus and synaptopathy is critical. At the same time, the screening should be testing different parts of the system while not being too extensive in time to avoid effects of fatigue.

The present study attempts to categorize a subclass of tinnitus sufferers with normal hearing thresholds. A screening procedure was developed, consisting of psychophysical and physiological measures hypothesized to be sensitive to the presence of tinnitus and cochlear synaptopathy.

The data collected were used as input for a clustering algorithm. The objective to apply a clustering algorithm were to check if the algorithm is able to discriminate between tinnitus and a control group, and to evaluate if application of this algorithm can identify subgroups in the group of tinnitus sufferers.

## Materials and methods

### Participants

Twenty listeners suffering from tinnitus participated in the experiment (tinnitus group, mean age 32.2 years, 6 females). An age (+/- 4 years, mean 0.73, std 1.34) and audiogram (+/- 10 dB per each frequency) matched group of 15 normal hearing listeners (30.2 mean age, 6 females) was included as control (control group). Inclusion criteria for the tinnitus group were a) non pulsatile, subjective persistent tinnitus (i.e. not transient as related to a temporal threshold shift or upper respiratory tract infection); b) audiometric thresholds not higher than 20 dB HL for the for the following audiometric frequencies: 125, 250, 1000, 2000, 3000, 4000, 6000, 8000 Hz. Each listener in the tinnitus group completed the Tinnitus Handicap Inventory (THI) questionnaire (mean: 22.2, min: 4, max: 54, standard deviation: 13.34). Otoscopy was performed to exclude obstruction by cerumen or infections. All participants provided informed consent and all experiments were approved by the Science-Ethics Committee for the Capital Region of Denmark (reference H-16036391).

### Apparatus

The psychoacoustical measures (high frequency audiometry, psychophysical tuning curves, tinnitus likeness, adaptive categorical loudness scaling) were implemented as custom software in MATLAB [22]. Stimuli were generated digitially, converted into an analogue waveform (RME Fireface UCX soundcard), amplified (SPL Phonitor MINI) and presented through headphones (Sennheiser HDA 200). The calibration was done by equalizing the transfer function (amplitude and phase) of the transducer using a FIR filter of order 2047. The transfer function was measured using the sound level meter NorSonic Nor139, the artificial ear: G.R.A.S. 43AA-S2 (ear simulator kit according to IEC 60318–1 & -2, with pre-polarized microphone) and the calibrator: Brüel & Kjær 4230 or 4231 sound calibrator. The wide band typanometry (WBT) and MEMR latencies were measured with the Interacoustics Research Platform and the Titan Suite software (Interacoustics A/S). The Titan research platform was used to measure the WBT and MEMR. The Titan Suite software was used to calculate the latencies.

### Tinnitus likeness

Tinnitus likeness was measured for all listeners in the tinnitus group. The loudness and pitch of the individual listeners’ tinnitus was measured using a tinnitus likeness (TL) procedure based on [23] using a) pure tones and b) 1/3rd octave wide narrow-band noise with Gaussian amplitude distribution as a probe. In a first step, the listeners were asked to adjust the level of a 1 kHz probe tone to match the loudness of the tinnitus. This was repeated three times. The average level of these three repetitions was used as the probe level for the frequency rating of the tinnitus in the second step. To match the pitch of the tinnitus, probes tones with frequencies between 0.125 kHz and 14 kHz were randomly presented for a duration of 2 s including 50 ms raised-cosine ramps at on- and offset. For each frequency, the listener was asked to rate the likeness of the probe with their tinnitus on a scale between 1 (“not similar at all to my tinnitus”) to 10 (“sounds exactly like my tinnitus”). The probe was repeated in intervals with 2 s silent intervals, until the participant did not give an answer. The listener was also given the option “not heard”. The maximum of the likeness spectrum was identified and used for loudness matching with tones in the third step: The listeners were asked to match the level of the probe centered at this frequency with the loudness of their tinnitus. This was repeated three times. The average level of these three repetitions was used in step four: To match the pitch of the tinnitus with the narrow-band noise probes, probes with the same center frequencies as in the second step were used.

### Adaptive categorical loudness scaling

Loudness growth was measured using an adaptive categorical loudness scaling (ACALOS) procedure [24] for all listeners in the control group and the tinnitus group. The stimulus consisted of a 1/3rd octave wide noise centered at the test frequency (0.25 kHz, 0.5 kHz, 1 kHz, 2 kHz, 4 kHz, 6 kHz) with a duration of 1000 ms including 50 ms raised-cosine on- and offset ramps. The frequencies were presented in random order and the listeners were asked to rate each sound from “Not heard” (corresponding to a value of 1) to “Extremely loud” (corresponding to a value of 50). The maximum level of the stimulus was set to 95 dB HL. In the the first trial, the level of the stimulus was set to 65 dB SPL. The next trial in a run was chosen dependent on the answer given in the previous sound presentation and a continuous function was fit through the measured data points to obtain the frequency specific loudness growth function. For details on the procedure, please see [24] and [25].

### Wideband tympanometry and middle ear muscle reflex

Wideband tymapanometry (WBT) and MEMR were measured ipsilaterally using the Titan system (Interacoustics A/S) and a custom software (Interacoustics Research Platform) implemented in [22] for all listsners in the control group and the tinnitus group. To measure the WBT the ear canal pressure was swept between -300 daPa and 200 daPa in descending and ascending direction with a speed of 100 or 300 daPa/s. The data were consistent for the different pump speeds in the same listener when tested in a subset of the listeners. The individual tympanometric peak pressure (TPP) was defined as the peak of the WBT and used for the pressurization before measuring the MEMR. The middle ear muscle reflex (MEMR) was measured using a paradigm based on [26] consisting of a series of clicks and activators (click-activator-click). The activator in this case was 506ms of white noise with onset and offset of 2.3 ms, respectively, obtained with a Kaiser window. The level of the noise was varied between 75 dB SPL and 105 dB SPL in steps of 5 dB. The transducer was calibrated based on peak-to-peak voltage of a 1 kHz tone. The strength of the reflex was quantified using the change in sound energy between the average of four stimuli with the elicitor and the baseline [27]. This will be referred to as DELTA absorbance. The threshold criterion for presence of the MEMR was chosen arbitrarily to be a DELTA absorbance of 0.03. The latency of the MEMR was recorded with the Titan suite software for pure tone probes with frequency of 500 Hz. The threshold used in this part of the experiment was calculated with the Titan Suite. The latency was calculated at a level of 10 dB above the reflex threshold subject to an upper limit of 100 dB SPL.

### Fast high-frequency audiometry, psychophysical tuning curves and tinnitus tuning curves

High-frequency audiometry (HFA), psychophysical tuning curves (PTC), and tinnitus tuning curves (TTC) were implemented using a Bayesian algorithm based on [28]. HFA and PTC were measured for all listsners in the control group and the tinnitus group. TTC were measured only for the listeners in the tinnitus group. The algorithm maximizes the information obtained from each estimate by selection of the measurement parameter to reduce the uncertainty based on the listeners responses. The parameters used in HFA, PTC and TMC are summarized in Table 1.

**Table 1:** Parameters for the Bayesian procedure applied to high-frequency audiometry (HFA), psychophysical tuning curve at 1 kHz (PTC1k), psychophysical tuning curve at the tinnitus frequency (PTCtf), and the tinnitus tuning curve (TTC). *f*_*T*_ indicates the tinnitus frequency.

	HFA	PTC1k	PTCtf	TTC
Start frequency (kHz)	12	1	*f*_*T*_	*f*_*T*_
Covariance	0.5	0.3	0.3	0.3
Silent trials	5	-	-	-
Min frequency (kHz)	8	0.5	*f*_*T*_/2	*f*_*T*_/2
Max frequency (kHz)	16	2	2*f*_*T*_	2*f*_*T*_
Max level (dB SPL)	90	90	90	90
Min level (dB SPL)	-10	-10	-10	-10
Nr. repetitions	3	2	2	1
Stimulus duration (ms)	250	500	500	2000

The HFA was implemented as a 1-interval-2-alternative forced choice (1-I-2-AFC) procedure. One stimulus interval of the HFA contained a series of three tone bursts, each with a duration of 250 ms including 20 ms on- and offset ramps and an inter-pulse gap of 100 ms. The tone bursts had frequencies between 8 kHz and 16 kHz. The listeners were asked to indicate if they heard the tone (“Yes”) or not (“No”).

The PTC was implemented as a 2-interval-2-alternative forced choice (2-I-2-AFC) procedure. For the PTC, the stimulus contained two noise bursts with a bandwidth of 0.5 octaves and Gaussian amplitude statistics. The noise bursts were generated in the frequency domain by setting everything outside the pass band to zero. The tone bursts were centered at 1 kHz (for the PTC at 1 kHz, PTC1k) or at the tinnitus frequency identified by the tinnitus likeness measure (TL). The probe for the PTC at tinnitus frequency (PTCtf) had a constant level corresponding to the loudness found in the tinnitus loudness matching. If that level was below threshold, the level was set to 5 dB above the value found in the loudness matching experiment. The same frequency and level used for a given listener from the tinnitus group was used for the matched listener in the control group. The probe for the PTC at 1 kHz, had a level of 10 dB above the individual threshold in quiet at 1 kHz. The center frequency of the noise was varied between +/-1 octaves around the the reference frequency. The listeners were asked if the two sounds were different.

The TTC was implemented as a 1-interval-2-alternative forced choice (1-I-2-AFC) procedure. For the TTC, the stimulus was a single 0.5 octave-wide noise burst with Gaussian amplitude distribution and a duration of 2000 ms including 20 ms on- and offset ramps. The noise was centered at the tinnitus frequency. The listeners were asked to indicate whether the tinnitus is audible (“Yes”) or not (“No”) in the presence of the noise.

### Clustering

The collected data were organized into a 35 x 18 matrix with (participant x measures). The MEMR was measured twice, but only one of the two was included in the processing. The decision was based on the WBT. The WBT had to be positive and have the maximum peak in the pressure range between -150 to 150 daPa. If both measurements met these criteria, the first measurement was used. In addition to the strength of the MEMR, the MEMR latency for a tone at 500 Hz was also included in the predictors. The PTCtfs were projected onto categorical outputs: U-shaped (USH), flat (F), other (O). The TTCs were projected onto the categories: USH, F, O or non-maskable (NM). The categorization was conducted by three independent judges, distributing the raw data into the provided categories. The ACALOS was quantified in a single number derived from the mean (through the various frequencies) of the difference between the most comfortable level and hearing threshold. The tonal/noisy and the tone likeness of the tinnitus are information retrieved through the tinnitus likeness test. The first indicate the type of tinnitus and the second the higher likeness rating for tones. The HFA was tested twice. The median of the two tests has been calculated separately and the mean of the two values was chosen as input for the clustering. Age, tinnitus type (monaural or binaural), and the Tinnitus Hearing Impairment score were obtained through direct communication and the questionnaire.

To determine the optimal number of clusters, the silhouette algorithm was used [29]. The silhouette calculates for each observation the ratio of the distance intra-cluster and inter-clusters. The closer the output value of the silhouette is to 1 the better is the clustering. The closer is to 0, the higher is the uncertainty about which cluster the observation belongs to. In this analysis the Gower distance was used. The Gower distance, or similarity, can be calculated for numerical, categorical, logical or text data. In particular, the distance between two observations would be the average of the feature-specific distances in the range [0 1]. If the feature is numeric, the distance will be the ratio between the difference of the two values and the maximum range for that feature. If the feature is categorical, the value will be 1 if the features fall in the same category, 0 otherwise.

After selecting the number of clusters, the algorithm Partitioning around Medoids (PAM) was applied to identify the clusters. The algorithm identifies *k* objects, medoids, defining the clusters, where *k* is the number of clusters. The medoids are always defined among the elements to cluster [30]. The strategy of the algorithm is to minimizes the distance between the object to classify and one of the medoids. The distance between the objects to classify and the medoid is given calculating the Gower distance [31].

### Random forest

Given the output of the clustering, the next step was to try to understand which features contributed the most to the classification. To be able to select the most informative predictors, a Random Forest algorithm [32] available in MATLAB [22] was used. First, a set of 500 decision tree weak learners has been generated and trained for regression with a Bagging method. Bagging, as well as Boosting, is a method that consists in training more models with the same learning algorithm randomly sampling data-points to generate training data sets for the weak learners [33]. We then used the ensemble regression function that combines the weak learner models with the data to improve the prediction accuracy of the learning. To avoid selecting the settings manually, a Bayesian Optimization (BO) was used to optimize the choice of the hyperparameters [34]. The parameters that were optimized are: method (either Bagging or LSboost), the number of cycles for which the ensemble is trained, the learning rate, and complexity. The importance of the different predictors was calculated for better interpretability of the clusters relative to the used dimensions. To maximize the reliability of the result this routine has been run 50 times and the importance factor evaluated as the mean of all outputs.

## Results

### Tinnitus likeness

The results of the Tinnitus Likeness were split into two categories: i) The rating of similarity of the participant’s tinnitus with the tone that they picked to be the most similar (TonLiken), and ii) the information whether the tinnitus was rated more similar to a tone or to noise (Ton/Nois). Out of 20 tinnitus participants five (s03, s09, s12, s14, s20) rated their tinnitus to be more similar to noise, twelve rated it to be closer to a tone, and three (s19, s15, s08) rated in the same way the noise and the tone. The TonLiken was very variable across participants ranging from 1.67 (s12) to 10 (s18) on a scale from 0 to 10. The participants whose ratings were below 6 were s01, s09, s12 and s20 and roughly overlap with the group which rated the noise to be more representative of their tinnitus. Participant s01, however could not find any sound which resembled their tinnitus. Individual data can be found in S1, S4 and S5 Figs. The majority of the tinnitus group rated their tinnitus to be more similar to high frequency sounds. Since the PTCtf test was performed presenting a tone whose frequency was supposed to be the most close to the tinnitus, the information for tones is reported. Nine out of 20 participants chose the most similar tone to their tinnitus to be at 14 kHz, two at 12.5 kHz, one at 10 kHz four at 8kHz, two at 6 kHz, one at 3 kHz, one at 500 Hz (Table 2).

**Table 2:** Summary of the tinnitus frequencies for each participant (IDs) assessed with the tinnitus likeness. In the PTCtf the control particpants (IDc) have been tested with the frequency of the participant matched.

IDs	IDc	TF
s01	c01	14 kHz
s02	-	10 kHz
s03	-	8 kHz
s04	c04	12.5 kHz
s05	c05	14 kHz
s06	c06	14 kHz
s07	-	14 kHz
s08	c08	14 kHz
s09	-	500 Hz
s10	c10	12.5 kHz
s11	c11	14 kHz
s12	-	3 kHz
s13	c13	14 kHz
s14	c14	8 kHz
s15	c15	8 kHz
s16	c16	6 kHz
s17	c17	6 kHz
s18	c18	14 kHz
s19	c19	8 kHz
s20	c20	14 kHz

### Adaptive categorical loudness scaling

**Fig 2:**
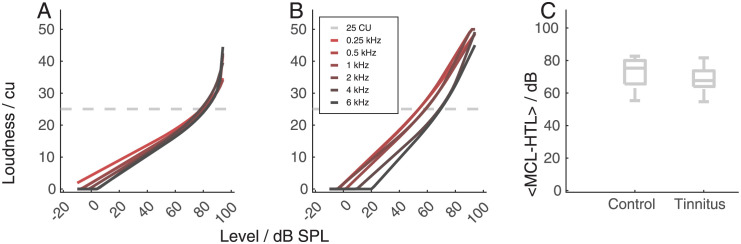
Panels A and B: ACALOS results for two exemplary participants. In panel A, the loudness growth function shows a fast growth for stimulus levels above around 80 dB SPL. The loudness growth functions for all frequencies cross the 25 CU line (most comfortable level) at similar stimulus levels. For the listener in panel B, there is a higher difference between loudness growth across frequency and, compared to the listener in panel A, a more linear growth across the tested input levels. Panel C shows the comparison of the data for the control group (left) and the tinnitus group (right). The difference between the two groups is not significant.

Fig 2(A) and 2(B) show two exemplary loudness growth functions obtained with adaptive categorical loudness scaling (ACALOS). Individual data can be found in S6 and S7 Figs. The loudness growth functions were quantified using the lower slope, the upper slope, and the breakpoint of the fitting function. The ACALOS was only performed until 95 dB HL. For some of the listeners the loudness perception didn’t reach the maximum rating (50) either for all the frequencies or a subset of them at this stimulus level. Hence, the mean of the difference between the most comfortable level (MCL) and the hearing threshold (HTL) across frequencies was used as an indicator for the presence of hyperacusis. The ACALOS rating ranged from 54.67 dB (s09) to 82.67 dB (c18). The median of the tinnitus group was 68.83 dB and the median of the control group was 75.33 dB. In line with the initial hypothesis, the most comfortable level was higher for the control group compared to the tinnitus group. But the difference was not statistically significant (Fig 2, panel C). Because no clear criteria exist, no binary classification (hyperacusis versus non-hyperacusis) was made before proceeding to the clustering and the MCL-HTL measure was used as input for the clustering.

### Wideband tympanometry and middle ear muscle reflex

**Fig 3:**
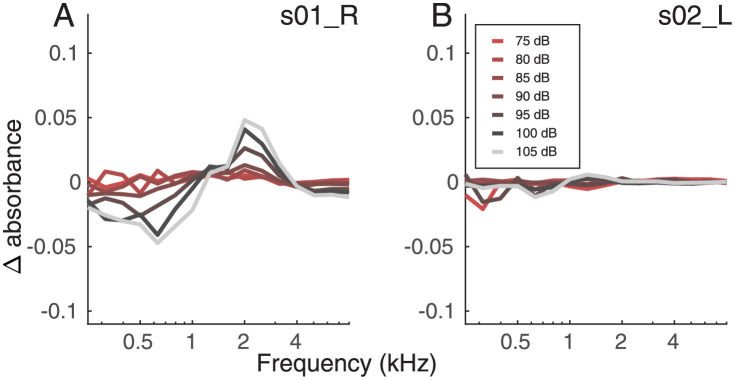
Exemplary results of MEMR in terms of delta absorbance in different frequency bands for listeners s01 (A) and s02 (B) in the tinnitus group. Each line represents a different level of stimulation from 75 to 105 dB SPL. The postfix to the listener label indicates the ear tested.

Fig 3 shows exemplary results for listeners with a strong (A) and weak (B) MEMR. Individual data can be found in S12 and S13 Figs. The MEMR strength was derived as the mean of the Delta Absorbance across frequency for each level for further analysis. In general, the MEMR showed a stronger response (grey) for high stimulus levels compared to low stimulus levels (red). The MEMR shows, however, high variability across listeners. For 105 dB SPL, for example, the minimum MEMR strength was 0.0324 (s13), while the maximum (not considering the outlier c20) was a factor of 20 higher with 0.6661 (c04). Similar patterns were found at the other levels. While some listeners had a strong response, others didn’t show any, even at higher levels (Fig 3 panel B). When comparing the tinnitus and control group, there was no significant difference at any level (Wilcoxon rank sum test, p > 0.05). The MEMR was tested twice, but only the less noisy result was kept. The MEMR for s11 resulted in too noisy data and was therefore discarded. One listener (s9) in the tinnitus group had a particularly high compliance of the tympanic membrane, therefore the WBT was tested with the clinical setting before testing the MEMR. The latency of the MEMR was calculated with the clinical setting of the Titan (Interacoustics A/S) at the frequency of 500 Hz. In the following analysis it will be referred to as ‘RL500’. The results for this variable ranged from 35 ms to 239 ms (mean 127.31 and standard deviation 51.60 ms). There was no significant difference between the tinnitus and the control group (Wilcoxon rank sum test, *p*> 0.05).

### Fast high-frequency audiometry, psychophysical tuning curves, and tinnitus tuning curves

**Fig 4:**
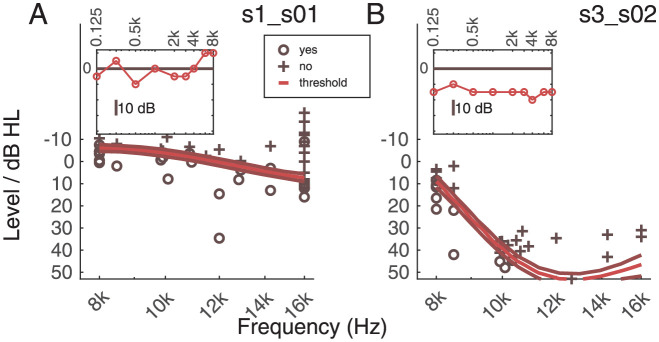
Two exemplary results of HFA for listeners s01 and s02 (tinnitus group). The insets show the results of the standard audiometry up to 8 kHz. The central lighter line represents the PTC. The two darker lines represent the limits of the one standard deviation shift. The circles represent the trials in which the target has been heard, and crosses represent the trials in which the target has not been heard.

Fig 4 shows two exemplary results for the HFA. Individual data can be found in S8–S11 Figs. The central lighter line represents the threshold obtained by an interpolation of the output of the Bayesian algorithm. The two darker lines represent the two extremes of the most likely probability to be within one SD in the 50% contour graph. The circles and the crosses represent the responses to the trials. Circles represent trials that have been heard (“yes” to the question “Did you hear the sound?”), and crosses represent sounds that have not been heard (“no” to the question “Did you hear the sound?”). The standard audiometry (125–8000 Hz) for the two listeners is presented in the inset. The two listeners in panels A and B represent the differences found in the group of audiometrically normal hearing listeners (i.e., in the range from 125 Hz to 8 k Hz). Some listeners showed thresholds <15 dB HL for the whole frequency range up to 16 kHz, while others showed a strong decline in sensitivity at frequencies higher than 8 kHz.

Across subjects, the HFA results showed high variability. The HFA was measured twice, and for each measurement the median across frequencies was calculated. The mean of the two medians was used for further analysis (‘MMedian’). The intersubject standard deviation of the mean of the two median values of the two HFA measurements was 13.99 (max:41.42 min:-4.22). This was expected since the age range of the participants varies from 22 to 60 years old. A strong correlation of the HFA with age was found (Spearman correlation, 0.74; p = e-7). The test-retest reliability of the median of the two measurements was quantified using the ICC measure [35, 36]. The repeatability of the HFA results was strong (ICC = 0.989). The median of the HFA measure for tinnitus listeners was 7.815 dB HL, while for controls was 3.25 dB HL. The difference was not statistically significant (Wilcoxon rank sum test, *p*> 0.05).

**Fig 5:**
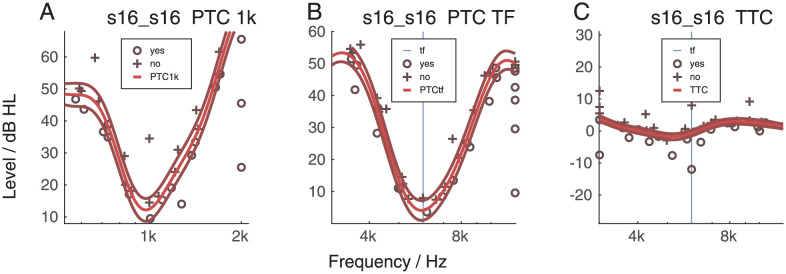
Panel A: The psychophysical tuning curve (PTC) at 1 kHz for listener s16 (tinnitus group). Panel B: For the same participant the PTC at the tinnitus frequency (tf), in this case 6 kHz. Panel C: For the same participant the tinnitus tuning curve (TTC).

Fig 5 shows the PTC at 1 kHz (PTC1k, panel A), the PTC at tinnitus frequency (PTCtf, panel B) and the tinnitus tuning curve (TTC, panel C) for the same listener. Individual data can be found in S2, S3, S14–S18 Figs. The vertical lines indicate the frequency of the probe frequency for the PTCtf and TTC derived from the the tinnitus (the output of the tinnitus likeness). This listener had a tuned response at the tinnitus frequency (panel B). For many subjects, however, the PTCtf was challenging, especially for those with tinnitus at very high frequencies (12,5 or 14 kHz). Therefore, the data above 12,5 kHz were considered unreliable and therefore excluded from the analysis. The PTCtf results have been classified either Tuned or Non-Tuned (T, NT) by three independent judges. Out of 35 participants, 15 had a tuned response (s01, s03, s04, s05, s10, s12, s14, s16, s17, s19, s20, c14, c15, c16, c18) and the rest had non-tuned response.

The TTC (panel C) was reported to be challenging especially by the participants describing their tinnitus as bilateral. All tests were conducted in one ear only. Hence, the masker was only provided in one ear. It is likely that the difficulty in the disassociation between the two ears made the test harder for this group of listeners. For each listener, the ear in which the tinnitus was subjectivity considered stronger was selected. If the tinnitus was self-reported as equally bothering, the choice was random. Participants reporting their tinnitus as central (s01, s07, s20) were considered in the same way as the one reporting it on both sides (s03, s04, s05, s06, s10, s11, s12, s13, s14, s17, s18, s19), taking into account a potential different behaviour of the TTC. Fewer participants had monoaural tinnitus (s02, s08, s09, s15, s16). This information was reported as ‘Mono/Bi’. The TTC results have been classified as Tuned, Non-Tuned or Non-Maskable (T, NT, NM) by three independent judges. Out of 20 tinnitus participants 4 resulted to have Non-Maskable tinnitus (s01, s02, s10, s20), 6 resulted to have Tuned TTC (s07, s13, s14, s17, s18, s19) and the rest Non-Tuned.

### Clustering

The results of the clustering differed depending on the inclusion or the exclusion of the tinnitus-specific predictors.

**Fig 6:**
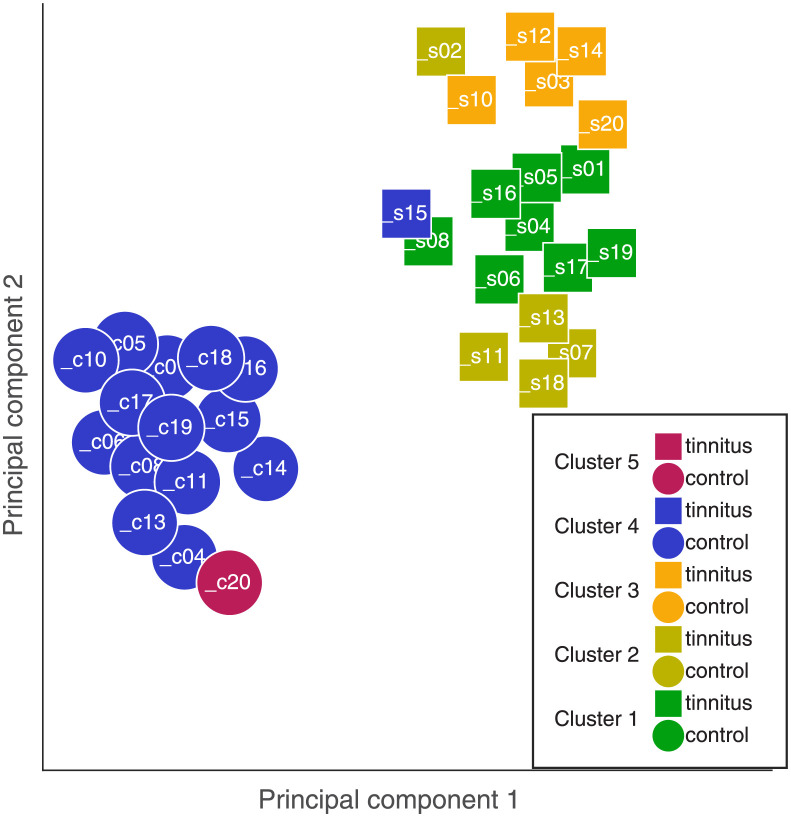
Clustering output when including all the 19 predictors and all the participants. The circles represent control participants, while the squares the tinnitus ones. Different colors represent different clusters.

The silhouette algorithm resulted in similar scores for 2 and 5 clusters for inclusion of all predictors. Hence, the clustering algorithm was performed with 2 and 5 clusters. The results for 2 clusters showed a clear separation of the tinnitus- and the control group (not shown). Of the tinnitus listeners, only subject s15 was included in the control group, despite suffering from tinnitus. The clear separation between the listener groups is anticipated because all predictors, including those exclusive to the tinnitus group, where available to cluster the data. Although trivial, this outcome shows the feasibility of the suggested clustering approach for the provided input data. The results for the 5 clusters (Fig 6) showed one outlier in the control group (c20) which presents the only element in Cluster 5. One cluster was found with all the remaining listeners from the control group plus one tinnitus participant (s15) as part of Cluster 4. The rest of the tinnitus group divided into 3 clusters (Cluster 1,2, and 3). It is interesting to note how tinnitus participants grouped in three different clusters. However, also in this case, since the clustering included tinnitus specific measures (Tinnitus Likeness, TTC, Mono/Bi, Ton/Nois) that were absent for controls (NaN) the separation between controls and tinnitus participants was anticipated.

To avoid biases due to the tinnitus-specific predictors, an additional clustering analysis was performed on the participants excluding the measures exclusive to the tinnitus group.

Based on the silhouette algorithm with the predictors excluding the tinnitus-specific parameters, the clustering algorithm was run with 3 clusters.

**Fig 7:**
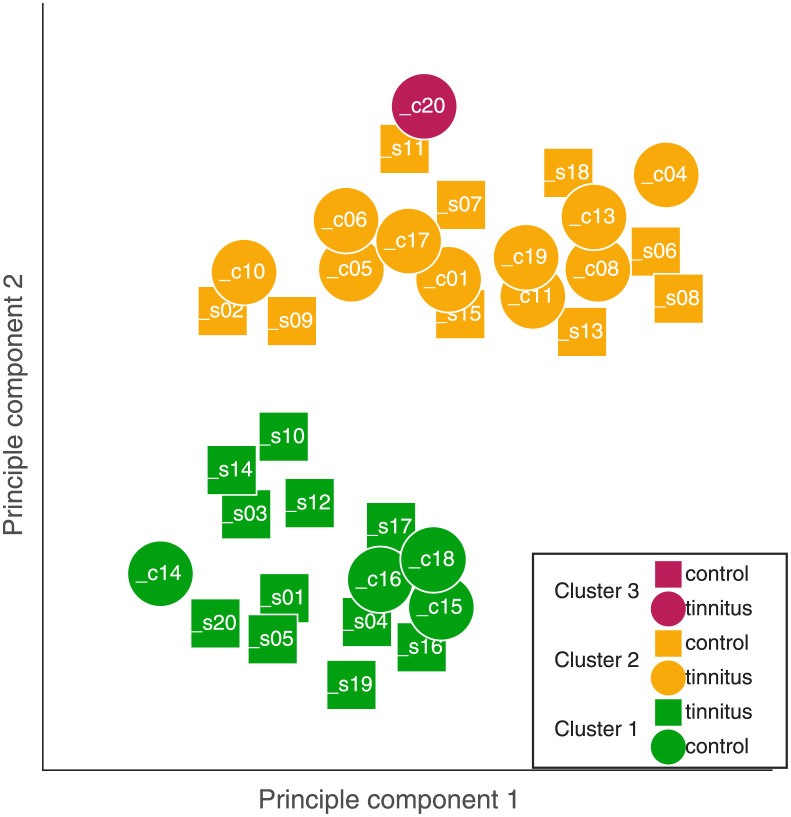
Same as Fig 6, but for clustering into three clusters and excluding tinnitus specific predictors.

The result of the clustering is shown in Fig 7. One cluster contained only the outlier c20, as in the clustering results with 5 clusters (see Fig 6). The other two clusters contain both control and tinnitus participants in different ratios. The first cluster contains the majority of the controls (10 out of 15) and about the half of the tinnitus participants (9 out of 20). The second cluster contains only few controls (4 out of 15) and about the half of the tinnitus participants (11 out of 20).

Table 3 shows the means of the numerical predictors and the categorical predictors for each cluster.

**Table 3:** Resulting parameter values of the clustering analysis with five (upper rows) and three (lower rows) clusters. For each cluster (Cl, first column), the following parameters are shown: HFA mean (dB HL), MEMR mean, ACALOS mean, age mean, RL500 mean (in ms), PTCtf, PTCtf summary, THI mean, TonLik mean, T/N summary, Mono/Bi (M/B) summary, and TTC summary. In the 5-cluster analysis, all measures were included, while in the 3-cluster analysis the tinnitus measures were excluded.

5-cluster analysis											
	**HFA**	**MEMR95**	**ACALOS**	**Age**	**RL500**	**PTCtf**	**THI**	**TonLik**	**T/N**	**M/B**	**TTC**
Cl1	1	0.22	68.33	28	91.5	6T+2NT	21.75	8.16	6T+2S	6B+2M	5NT+2T+1NM
Cl2	15.83	0.15	71.47	34.8	199	5NT	17.2	8.60	5T	4B+1M	3T+1NT+1NM
Cl3	24.25	0.09	66.83	40.5	115	5T+1NT	29.33	5.72	5N+1T	5B+1M	3NT + 2NM+1T
Cl4	9.30	0.17	72.78	30.07	144.5	11NT+4T	NA	NA	NA	NA	NA
Cl5	2.5	1.25	81.67	30	141	NT	NA	NA	NA	NA	NA
3-cluster analysis											
	**HFA**	**MEMR95**	**ACALOS**	**Age**	**RL500**	**PTCtf**	**THI**	**TonLik**	**T/N**	**M/B**	**TTC**
Cl1	9.45	0.19	71.38	33.33	114	15T	-	-	-	-	-
Cl2	12.12	0.14	69.79	31.16	142	19NT	-	-	-	-	-
Cl3	2.5	1.25	81.67	30	141	NT	-	-	-	-	-

For the 5-cluster analysis, clusters 1–3 (which contained all but one listeners from the tinnitus group) varied mainly in terms of HFA, MEMR95, RL500 and the PTCtf. There is consistency that the on average youngest listeners (Cl1) had the lowest HFA thresholds and the strongest MEMR95. The on average oldest listeners (Cl3) had the highest HFA and the the weakest MEMR95. The longest latencies RL500 were found in Cl2 which contained exclusively listeners with exclusively non-tuned PTCtf. Clusters 4–5 contained all listeners from the control group plus one listeners reporting tinnitus (s15). Cluster 5 contained only one listener (c20). All values for Cl4 were within the range of clusters 1–3 which indicates that it was the absence of the tinnitus-specific predictors (THI, TonLi,, T/N, M/B, TTC) which was underlying the separation of the clusters.

For the 3-cluster analysis, cluster 3 contained a single listener (C20). The main differences between clusters 1 and 2 were the PTCtf with only tuned and non-tuned characteristics, respectively. Listeners in Cl2 also had longer RL500 latencies compared to the listeners in Cl1. The main differences between clusters 1–2 and cluster 3 are the low HFA for c20 paired with a strong MEMR95.

Overall, the listeners could be grouped in to clearly separable clusters. But even for a relatively low number of clusters (3 and 5), the number of listeners in each group became relatively low. Hence, the small sample size might have contributed to the heterogeneity of the members (control / tinnitus) in each cluster.

### Random forest

**Fig 8:**
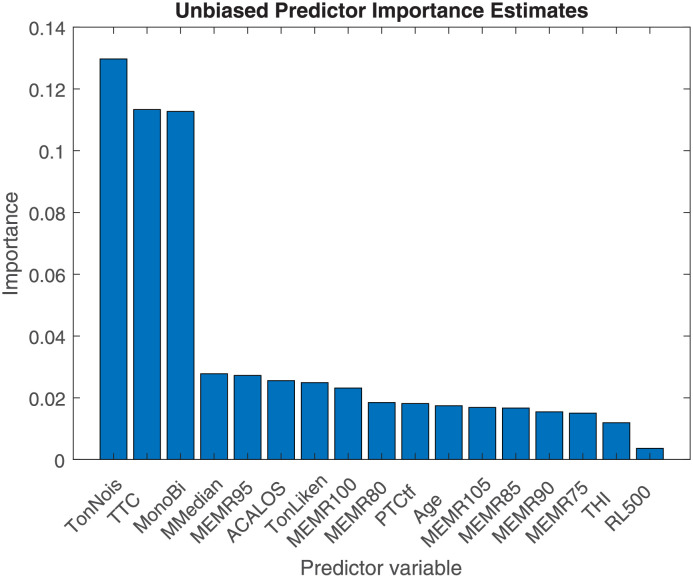
The importance analysis for the clustering results in 6.

The analysis of importance (Fig 8) showed that for the five clusters analysis the most important predictors were: tonal or noisy tinnitus (0.1297), TTC (0.1133), and monaural or binaural tinnitus (0.1127). The next factors had an importance of around 0.03 and were more variable, despite representing the mean of 50 repetitions of the analysis. Thesese factors included HFA (MMedian), MEMR at 95 dB (MEMR95), ACALOS and Likeness of tinnitus to a tone (TonLiken). The three most important measures were in the Only-Tinnitus category, that means applicable only to the tinnitus group. Not surprisingly, the most important measures are tinnitus-only measures. These easily separate the control (that lack these information) from the tinnitus group. However, The TonLiken predictor is not among the highest rated, but also tinnitus related.

**Fig 9:**
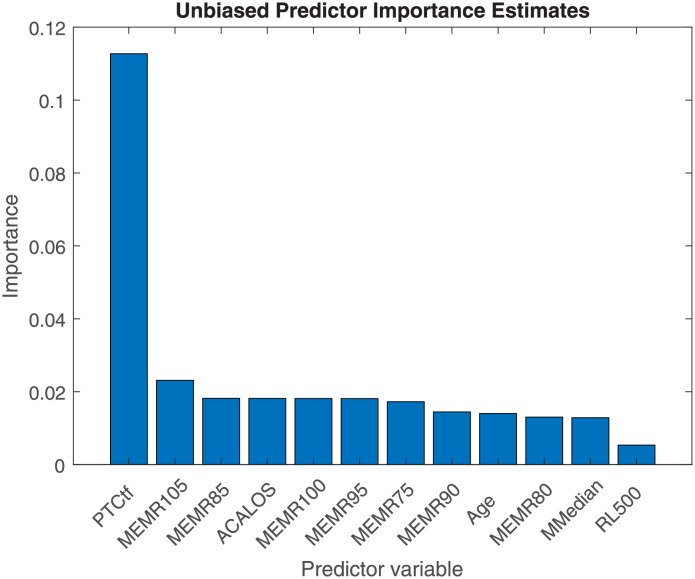
Same as Fig 8, but for the clusters in shown in Fig 7.

The importance analysis with the clustering without tinnitus specific measures is shown in Fig 9. In this analysis the PTCtf is the most important predictor (0.1127) and all the others except the latency of the reflex on a comparable level (around 0.02). The maximum level of Importance, however, for both cases is around 0.1–0.13.

## Discussion and conclusions

In the present study, various data were collected with the goal to have multiple sources of variability as input into a cluster analysis. The test groups were compiled based on characteristics as homogeneous as possible within the two groups (tinnitus and controls) but also between the groups to reduce noise in the data. The analysis showed no significant difference between the tinnitus and the control group.

According to our hypothesis, a group of participants should have shown some characteristics usually linked to cochlear synaptopathy (CS). Our hypothesis related to CS was: i) increased thresholds at high frequencies [20], ii) decreased activation of the MEMR [26], iii) a lower MCL. Considering the first cluster with five groups, the cluster showing this characteristics was cluster 3. Interestingly, cluster 3 was also the one with higher average age (Table 3). The type of tinnitus was mainly tonal for the first two clusters and noise-like for the cluster whose characteristics were closer to CS. Similarly, evidence exist that noise-like tinnitus can be more common in older patients with higher-level hearing loss [37]. Also the mean of the THI questionnaire responses was higher for Cluster 3. However, the three tinnitus groups were relatively small (Cluster 1 = 8 participants, Cluster 2 = 5 participants, Cluster 3 = 6 participants). Due to the small size of the clusters, no statistical analysis was run to assess the significance of these differences.

We hypothesized that participants with noise-like tinnitus would show a different PTCtf compared to the tinnitus participants with tonal tinnitus. In fact, the tonal tinnitus perception could be a distraction when trying to distinguish a trial with or without probe tone. The results showed that cluster 1 and 3 were the ones with mainly tuned (T) PTCtf, which we can consider to be consistent with PTC in normal hearing listeners. Cluster 1 was composed of participants having mainly tonal tinnitus, while cluster 3 was mainly composed of listeners with noise-like tinnitus. Therefore, the first result was not in agreement with the initial hypothesis. In addition to this, Listeners in Cluster 3 had a mean tinnitus frequency lower that the ones in Cluster 1 (7.67 kHz for Cluster 3, 11.06 kHz for Cluster 1), which made the task even harder. Listeners reported the task to be more demanding with higher frequencies when compared with the same test performed at 1 kHz. Cluster 2 and 3 are in line with the hypothesis that PTCtf shows a shape similar to non-tinnitus sufferers for noisy tinnitus than for tonal tinnitus. This is, however, inconsistent with Cluster 1.

For what concerns the monaural or binaural characteristic of tinnitus, the distribution was uniform across the three groups, so there was no group having a single output for this predictor. We hypothesized that TTC would have been mainly Non-tuned (NT) or Non-maskable (NM) for binaural tinnitus but it is not the case from the data, although participants with binaural tinnitus reported the high difficulty in disentangling the perception in the two ears.

**Fig 10:**
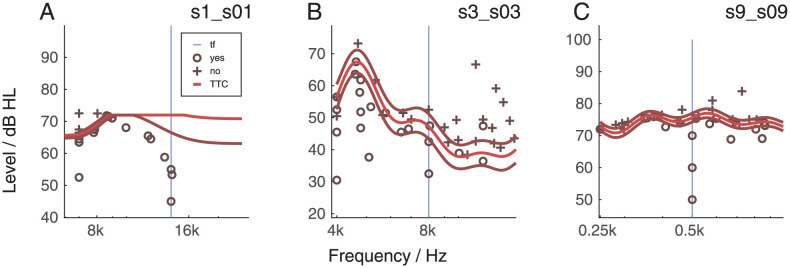
Tinnitus Tuning Curve (TTC) for three exemplary participants: s01 (panel A). s03 (panel B), s09 (panel C). The y-axis shows the level of the masker. The x-axis shows the frequency tested. s01 reported that the tone most similar to his tinnitus had a frequency of 14 kHz, s03 a frequency of 8 kHz and s09 a frequency of 500 Hz.

It needs to be underlined that in this experiment HFA, PTC and TTC were performed with a Bayesian procedure. This allowed to decrease the duration of the experiment and to get a continuous result. However, the PTC at very high frequency was perceived as a very hard task, giving noisy results. Therefore, we decided to handle the results of the last two tests (PTC, TTC) grouping them into categories and this might have created some bias in the clustering. Exemplary outcomes of the PTC are shown in Fig 10. Panel A indicates the unreliability of the data for listeners with a high-pitched tinnitus. Panel B illustrates a listener where the tuning of the PTC likely seemed to be affected by the presence of the tinnitus. Panel C illustrates a listener where tinnitus frequency and PTC seemed independent from each other. These data illustrate the strong variability in this outcome measure. An automated procedure with a higher number categories might refine the clustering result, but will require a much higher number of listeners for the analysis.

In the second clustering we see, in fact, that the PTCtf takes over the clustering. However, the TTC and PTC can give interesting insights about the maskability of the tinnitus and the tuning of the cochlea, respectively, and shed light on the hearing mechanisms connected to tinnitus. The second clustering without tinnitus measures is less informative and the mean differences across groups are not very broad (Table 3). However, what can be extrapolated from this result is that tinnitus participants had no homogeneous and distinct features with respect to the controls. With more participants we would have expected to see a mixed group with tinnitus and controls having characteristics leaning towards CS.

The reason for the different findings across studies might lie in the differences how the measures were assessed. One study [18] measured MEMR using a threshold criterion of “…reduction in compliance of 0.02ml or greater with appropriate morphology and no evidence of significant measurement artifact” [18] using a clinical paradigm with a tonal probe and coupler calibration. Another study [17] and the present study used a broadband elicitor and an in-situ calibration method. [18] and the present study used ipsilateral elicitors, while [17] used contralateral elicitors. The metric used in [18] was a threshold, while [17] and the present study used a metric reflecting the MEMR strength. In [18] and in the present study, listener were assessed with audiometry up to 14 kHz (16 kHz in the present study), while [17] only screened the audiogram up to 8 kHz and included listeners in the tinnitus group up to 30 dB HL. In the present study, no significant differences were found in MEMR strength between the tinnitus group and the control group at any level measured (Wilcoxon rank sum test, p > 0.05). The authors in [17] argue that their paradigm reduced the influence of the medial olivocochlear (MOC) reflex by the low rate of click presentation. The paradigm used in [18] and in the present study is, however, more consistent with the most sensitive measure of synaptopathy in mouse [38]. Hence, it can not be excluded that different variations of MEMR strength variability across listeners and mixed effects of MOC reflex and high-frequency sensitivity loss could have an effect on the results. While the studies by [17] and [18] rely on a pairwise comparison of outcome measures, the grouping of the elements in the high-dimensional predictor space into clusters might be robust against the introduced variability and more sensitive to consistencies in correlations across multiple predictors. This benefit might, however, be jeopardized by the need for larger sample numbers to allow proper statistical estimates of the distributions of the data.

There might be additional factors contributing to the creation of clusters in the present study. One factor not explicitly controlled for is gender of the listeners. Previous studies on gender effects on comorbidities of tinnitus found none of weak correlations between gender and suggested tinnitus-related comorbidities. A recent study evaluated tinnitus severity using THI along with multiple indicators of psychiatric distress in a sample of 245 listeners [39]. They found no gender effect on tinnitus severity in their sample, but weak correlations of gender and a number of psychiatric conditions like depression (females) or stress (males). Another study [40] found similar results in a sample of 107 listeners with correlations only between gender and psychiatric comorbidities, but not direct measures of tinnitus like THI. The sample size in the present size is considerably lower and does not allow for a statistical analysis of similar correlations. However, the numbers in the various clusters were rather balanced and indicate no trend towards an impact of gender on the results (11/4 and 11/8 male/female in the 3-cluster analysis in the two clusters containing more than one listener). It might be interesting to evaluate putative correlations between the measures used in the present study and psychiatric diseases by addition of another dimension in the clustering analysis.

Subject 15 (s15) became an outlier in the 5-cluster analysis. It was assigned into a cluster with listeners from the control group, despite reporting tinnitus. Even though only a individual listener, this results warrants some speculation. Based on the results, it is challenging to link the assignment of the listener to similarities to the control group. But it might be interesting to speculate about the exclusion of this listener from the tinnitus-dominated cluster, despite its proximity in the projection on the first two principle components. The listener reported a broadband tinnitus (TL, panel O), similar to a high number of other listeners in this group. It showed a relatively flat HFA below (20 dB HL in the measured range) which was highly similar to a large number of other listeners in the group. Also loudness perception (ACALOS) and the psychophysical tuning at 1 kHz (PTC1k) seemed similar to a high number of other listeners. This listener differed from the other listeners in the tinnitus group in terms of the MEMR. Compared to most other listeners showed the MEMR of s15 (panel N) a relatively flat profile with little variation with intensity. At low frequencies, there is a tendency towards a rather constant delta-absorbance which is different to most other listeners. for other listeners, the delta absorbance tends to be more negative between 0.5 kHz and 1 kHz compared to frequencies below 0.5 kHz. Only at the highest level measured (105 dB) was the MEMR more in agreement with that of the other listeners with negative values up to about 1 kHz and positive values above 1 kHz. The psychophysical tuning curve at tinnitus frequency (PTCtf) showed a, compared to the other listeners, relatively shallow U-shape. Also in the tinnitus tuning curve (TTC, panel M), this listeners showed a concave shape with a maximum at the tinnitus frequency. The relatively unique shape of the MEMR indicates a reduced sound level sensitivity of the mechanism modifying the sound transduction into the cochlea. This mechanism was only visible in the data at the highest level measures. The PTCtf and the TTC might suggest that there is an interaction of the acoustic probe with the tinnitus mechanism. In the PTCtf, the U-shape seems broadened due to the presence of the tinnitus. In the TTC, the concave shape and the peak at the tinnitus frequency might be a sign for entrainment of the tinnitus frequency to the external probe tone. All these indications are derived from the data of a single listener. It might be interesting to confirm similarity across these measures in a group of listeners selected based on a single of the measures. It needs, however, to be noted that the relatively small sample size might lead to a poor estimate of the high-dimensional distributions derived in the predictor space. This undersampling might hence lead to noise in the assignment to clusters and hence the appearance of this outlier.

In conclusion, the use of clustering for the categorization of tinnitus participants revealed that tinnitus participants with normal hearing can still have very different outputs in many tests. Confirming, in other words, that tinnitus is a very diverse phenomenon for causes, comorbidity and hearing disorders. However, the value of the silhouette function, showing the validity of the classification, was not very high. This means that to confirm the results of this preliminary study a higher number of participants needs to be recruited. Finally, clustering with a big data set, potentially including dimensions of psychiatric comorbidities could be the starting point towards a refined categorization of tinnitus.

## Supporting information

S1 Fig Representative tinnitus likeness results for three listeners. The figure represents the Tinnitus Likeness (TL) for participants s01 (panel A), s02 (panel B), s03 (panel C) and s04 (panel D). On the y-axis are reported the ratings of similarity from 1 to 10 of each frequency. The frequencies rated are in the x-axis (250 Hz, 500 Hz, 1 kHz, 2 kHz, 3 kHz, 4 kHz, 6 kHz, 8 kHz, 10 kHz, 12.5 kHz, 14 kHz). Each frequency is rated three times.
(EPS)

S2 Fig Exemplary results for the psychophysical tuning curve at 1 kHz. The figure represents the Psychophysical Tuning Curve (PTC) at 1 kHz for two participants, one belonging to the control group (c01, panel A) and one belonging to the tinnitus group (s01, panel B). On the y-axis is reported the level of the masker, while on the x-axis is reported the frequency of the masker. The target is a tone of frequency 1 kHz and level 5 dB higher than threshold. The central lighter line represents the PTC. The two darker lines represent the limits of the one standard deviation shift. The circles represent the trials in which the target has been heard, and crosses represent the trials in which the target has not been heard.
(EPS)

S3 Fig Exemplary results for the psychophysical tuning curve at tinnitus frequency. The figure represents the Psychophysical Tuning Curve (PTC) at tinnitus frequency for three exemplary participants. The frequency tested for the control participants was the one of the tinnitus of the participant they were matching. In panel A the control participant c01, tested at 14 kHz. In panel B the tinnitus participant s01, tested at 14 kHz. In panel C the tinnitus participant s09, tested at 500 Hz. On the y-axis is reported the level of the masker, while on the x-axis is reported the frequency of the masker.
(EPS)

S4 Fig Individual results of tinnitus likeness for tonal probes (tinnitus group). The figure represents the individual results of the Tinnitus Likeness (TL) for all the participants (from s01 to s20 panels A-T) for tones. On the y-axis are reported the ratings of similarity from 1 to 10 of each frequency. The frequencies rated are in the x-axis (250 Hz, 500 Hz, 1 kHz, 2 kHz, 3 kHz, 4 kHz, 6 kHz, 8 kHz, 10 kHz, 12.5 kHz, 14 kHz). Each frequency is rated three times.
(EPS)

S5 Fig Individual results of tinnitus likeness for noise probes (tinnitus group). The figure represents the individual results of Tinnitus Likeness (TL) for all the participants (from s01 to s20, panels A-T) for noises. On the y-axis are reported the ratings of similarity from 1 to 10 of each frequency. The frequencies rated are in the x-axis (250 Hz, 500 Hz, 1 kHz, 2 kHz, 3 kHz, 4 kHz, 6 kHz, 8 kHz, 10 kHz, 12.5 kHz, 14 kHz). Each frequency is rated three times.
(EPS)

S6 Fig Individual results for adaptive categorial loudness scaling (tinnitus group). The figure represents the individual results of the Adaptive Categorical Loudness Scaling (ACALOS) for all the tinnitus participants (from s01 to s20, panels A-T). On the y-axis the rating from 0 to 50 of the sound. On the x-axis the level of the sound presented. Each colored line represents a different frequency (250 Hz, 500 Hz, 1 kHz, 2 kHz, 4 kHz, 6 kHz). The horizontal dotted grey line represents the Most Comfortable Level (MCL) rated as with 25 out of 50.
(EPS)

S7 Fig Individual results for adaptive categorial loudness scaling (control group). The figure represents the individual results of the Adaptive Categorical Loudness Scaling (ACALOS) for all the control participants (from c01 to c20, panels A-O). The numeric part of the labels for each participant are equal to the labels of the tinnitus participant matched (in hearing loss and age). On the y-axis the rating from 0 to 50 of the sound. On the x-axis the level of the sound presented. Each colored line represents a different frequency (250 Hz, 500 Hz, 1 kHz, 2 kHz, 4 kHz, 6 kHz). The horizontal dotted grey line represents the Most Comfortable Level (MCL) rated as with 25 out of 50.
(EPS)

S8 Fig Individual results for high frequency audiometry (tinnitus group) for listeners s1-s10. The figures represent the individual results of the High Frequency Audiometry (HFA) for the tinnitus listener (from s01 to s10). Each participant has been tested twice except participant s17, which was struggling with the task. Each label on the top right of each panel is composed by two parts: the first is an incremental index and the second one is the label for the specific participant. On the y-axis are presented the hearing thresholds and on the x-axis the frequencies (from 8 kHz up to 16 kHz). The central lighter line represents the threshold. The two darker lines represent the limits of the one standard deviation shift. Circles represent trials that have been heard, and crosses represent sounds that have not been heard.
(EPS)

S9 Fig Individual results for high frequency audiometry (tinnitus group) for listeners s11-s20. Same as S8 Fig but for listeners s11-s20.
(EPS)

S10 Fig Individual results for high frequency audiometry (control group) for listeners c1-c10. The figures represent the individual results of the High Frequency Audiometry (HFA) for the tinnitus listener (from s01 to s10). Each participant has been tested twice except participant s17, which was struggling with the task. Each label on the top right of each panel is composed by two parts: the first is an incremental index and the second one is the label for the specific participant. On the y-axis are presented the hearing thresholds and on the x-axis the frequencies (from 8 kHz up to 16 kHz). The central lighter line represents the threshold. The two darker lines represent the limits of the one standard deviation shift. Circles represent trials that have been heard, and crosses represent sounds that have not been heard.
(EPS)

S11 Fig Individual results for high frequency audiometry (control group) for listeners 11-20. Same as S10 Fig but for listeners c11-c20.
(EPS)

S12 Fig Individual results for the middle ear muscle reflex (tinnitus group). The figure represents the individual results of the Middle Ear Muscle Reflex (MEMR) for all the tinnitus participants except s11, whose results were too noisy to be added. The panels (A-S) represent the participants s01 to s20 incrementally. On the y-axis of each panel is reported the Delta Absorbance, while on the x-axis the frequencies. Each line represents a different level of the sound tested, from 75 dB SPL to 105 dB SPL.
(EPS)

S13 Fig Individual results for the middle ear muscle reflex (control group). The figure represents the individual results of the Middle Ear Muscle Reflex (MEMR) for all the control participants. The panels (A-O) represent the participants c01 to c20 incrementally. The numeric part of the labels for each participant are equal to the labels of the tinnitus participant matched (in hearing loss and age). In each panel on the y-axis of each panel is reported the Delta Absorbance, while on the x-axis the frequencies. Each line represents a different level of the sound tested, from 75 dB SPL to 105 dB SPL.
(EPS)

S14 Fig Individual results for psychophysical tuning curve at 1 kHz (tinnitus group). The figure represents the individual results of Psychophysical Tuning Curve (PTC) at 1 kHz for all the tinnitus participants (from s01 to s20, panel A-R). The results for participants s17 and s19 are missing due to technical problems. In each panel on the y-axis is reported the level of the masker, while on the x-axis is reported the frequency of the masker. The target is a tone of frequency 1 kHz and level 5 dB higher than the individual threshold. The central lighter line represents the PTC. The two darker lines represent the limits of the one standard deviation shift. The circles represent the trials in which the target has been heard, and crosses represent the trials in which the target has not been heard.
(EPS)

S15 Fig Individual results for psychophysical tuning curve at 1 kHz (control group). The figure represents the individual results of the Psychophysical Tuning Curve (PTC) at 1 kHz for all the control participants (from c01 to c20, panel A-R). The numeric part of the labels for each participant are equal to the labels of the tinnitus participant matched (in hearing loss and age). In each panel on the y-axis is reported the level of the masker, while on the x-axis is reported the frequency of the masker. The target is a tone of frequency 1 kHz and level 5 dB higher than the individual threshold. The central lighter line represents the PTC. The two darker lines represent the limits of the one standard deviation shift. The circles represent the trials in which the target has been heard, and crosses represent the trials in which the target has not been heard.
(EPS)

S16 Fig Individual results for psychophysical tuning curve at tinnitus frequency (tinnitus group). The figure represents the individual results of the Psychophysical Tuning Curve at the tinnitus frequency (PTCtf)found with the Tinnitus Likeness (TL) for all the tinnitus participants. The results for participants s07 and s13 are missing due because of difficulties in performing the experiment. In each label on the y-axis is reported the level of the masker, while on the x-axis is reported the frequency of the masker. The central lighter line represents the PTC. The two darker lines represent the limits of the one standard deviation shift. The circles represent the trials in which the target has been heard, and crosses represent the trials in which the target has not been heard.
(EPS)

S17 Fig Individual results for psychophysical tuning curve at tinnitus frequency (control group). The figure represents the individual results of the Psychophysical Tuning Curve at tinnitus frequency (PTCtf) for all the control participants. The frequency tested for the control participants was the one of the tinnitus of the participant they were matching. The numeric part of the labels for each participant are equal to the labels of the tinnitus participant matched (in hearing loss and age). In each label on the y-axis is reported the level of the masker, while on the x-axis is reported the frequency of the masker. The central lighter line represents the PTC. The two darker lines represent the limits of the one standard deviation shift. The circles represent the trials in which the target has been heard, and crosses represent the trials in which the target has not been heard.
(EPS)

S18 Fig Individual results for the tinnitus tuning curve. The figure represents the individual results of the Tinnitus Tuning Curve (TTC) for all the tinnitus participants. The results for participants s02, s10 and s20 are not reported since they indicated they could still hear their tinnitus at any level presented. In each panel, on the y-axis is reported the level of the masker, while on the x-axis the frequency tested. The blue vertical line indicates the frequency indicated as most close to the tinnitus. The central lighter line represents the TTC. The two darker lines represent the limits of the one standard deviation shift. The circles represent the trials in which the participant could still hear the tinnitus, and crosses represent the trials in which the tinnitus was not heard.
(EPS)

## References

[1] McCormack A, Edmondson-Jones M, Somerset S, Hall D. A systematic review of the reporting of tinnitus prevalence and severity. Hearing Research. 2016;337:70–79. doi: 10.1016/j.heares.2016.05.009 27246985

[2] Association AT. Understanding the Facts;. Available from: https://www.ata.org/understanding-facts.

[3] McFerran DJ, Stockdale D, Holme R, Large CH, Baguley DM. Why is there no cure for tinnitus? Frontiers in Neuroscience. 2019;13:802. doi: 10.3389/fnins.2019.00802 31447630 PMC6691100

[4] Baracca G, Del Bo L, Ambrosetti U. In: Møller AR, Langguth B, De Ridder D, Kleinjung T, editors. Tinnitus and Hearing Loss. New York, NY: Springer New York; 2011. p. 285–291. Available from: 10.1007/978-1-60761-145-5_35.

[5] Crummer RW, Hassan GA. Diagnostic Approach to Tinnitus. American Family Physician. 2004;69(1):120–126+127–128. 14727828

[6] National Research Council (US) Committee on Hearing, Bioacoustics, and Biomechanics. Tinnitus: Facts, Theories, and Treatments. Washington (DC): National Academies Press (US); 1982. 25032460

[7] Weiler EWJ, Brill K, Tachiki KH. Quantitative electroencephalography and tinnitus: A case study. International Tinnitus Journal. 2000;6(2):124–126. 14689629

[8] Schaette R, McAlpine D. Tinnitus with a Normal Audiogram: Physiological Evidence for Hidden Hearing Loss and Computational Model. Journal of Neuroscience. 2011;31(38):13452–13457. doi: 10.1523/JNEUROSCI.2156-11.2011 21940438 PMC6623281

[9] Gu JW, Herrmann BS, Levine RA, Melcher JR. Brainstem auditory evoked potentials suggest a role for the ventral cochlear nucleus in tinnitus. Jaro—Journal of the Association for Research in Otolaryngology. 2012;13(6):819–833. doi: 10.1007/s10162-012-0344-1 22869301 PMC3505586

[10] Milloy V, Fournier P, Benoit D, Noreña A, Koravand A. Auditory brainstem responses in tinnitus: A review of who, how, and what? Frontiers in Aging Neuroscience. 2017;9:237. doi: 10.3389/fnagi.2017.00237 28785218 PMC5519563

[11] Hofmeier B, Wolpert S, Aldamer ES, Walter M, Thiericke J, Braun C, et al. Reduced sound-evoked and resting-state BOLD fMRI connectivity in tinnitus. Neuroimage: Clinical. 2018;20:637–649. doi: 10.1016/j.nicl.2018.08.029 30202725 PMC6128096

[12] Lanting CP, De Kleine E, Langers DRM, Van Dijk P. Unilateral tinnitus: Changes in connectivity and response lateralization measured with fMRI. Plos One. 2014;9(10):e110704. doi: 10.1371/journal.pone.0110704 25329557 PMC4203817

[13] Brotherton H, Plack CJ, Maslin M, Schaette R, Munro KJ. Pump up the volume: Could excessive neural gain explain tinnitus and hyperacusis? Audiology and Neurotology. 2015;20(4):273–282. doi: 10.1159/000430459 26139435

[14] Lobarinas E, Salvi R, Ding D. Selective Inner Hair Cell Dysfunction in Chinchillas Impairs Hearing-in-Noise in the Absence of Outer Hair Cell Loss. JARO—Journal of the Association for Research in Otolaryngology. 2016;17(2):89–101. doi: 10.1007/s10162-015-0550-8 26691159 PMC4791417

[15] Kujawa SG, Liberman MC. Synaptopathy in the noise-exposed and aging cochlea: Primary neural degeneration in acquired sensorineural hearing loss. Hearing Research. 2015;330:191–199. doi: 10.1016/j.heares.2015.02.009 25769437 PMC4567542

[16] Wu PZ, Liberman LD, Bennett K, de Gruttola V, O’Malley JT, Liberman MC. Primary Neural Degeneration in the Human Cochlea: Evidence for Hidden Hearing Loss in the Aging Ear. Neuroscience. 2019;407(Sp. Iss. SI):8–20. doi: 10.1016/j.neuroscience.2018.07.053 30099118 PMC6369025

[17] Wojtczak M, Beim JA, Oxenham AJ. Weak Middle-Ear-Muscle Reflex in Humans with Noise-Induced Tinnitus and Normal Hearing May Reflect Cochlear Synaptopathy. Eneuro. 2017;4(6):ENEURO.0363–17.2017. doi: 10.1523/ENEURO.0363-17.2017 29181442 PMC5702873

[18] Guest H, Munro KJ, Plack CJ. Acoustic Middle-Ear-Muscle-Reflex Thresholds in Humans with Normal Audiograms: No Relations to Tinnitus, Speech Perception in Noise, or Noise Exposure. Neuroscience. 2019;407(Sp. Iss. SI):75–82. doi: 10.1016/j.neuroscience.2018.12.019 30579832

[19] Fournier P, Wrzosek M, Paolino M, Paolino F, Quemar A, Noreña AJ. Comparing Tinnitus Tuning Curves and Psychoacoustic Tuning Curves. Trends in Hearing. 2019;23:1–16. doi: 10.1177/2331216519878539 31588855 PMC6783663

[20] Liberman MC, Epstein MJ, Cleveland SS, Wang H, Maison SF. Toward a differential diagnosis of hidden hearing loss in humans. Plos One. 2016;11(9):e0162726. doi: 10.1371/journal.pone.0162726 27618300 PMC5019483

[21] Möhrle D, Hofmeier B, Amend M, Wolpert S, Ni K, Bing D, et al. Enhanced Central Neural Gain Compensates Acoustic Trauma-induced Cochlear Impairment, but Unlikely Correlates with Tinnitus and Hyperacusis. Neuroscience. 2019;407(Sp. Iss. SI):146–169. 30599268 10.1016/j.neuroscience.2018.12.038

[22] MATLAB. version 7.10.0 (R2017a). Natick, Massachusetts: The MathWorks Inc.; 2016.

[23] Norena A, Micheyl C, Chéry-Croze S, Collet L. Psychoacoustic characterization of the tinnitus spectrum: Implications for the underlying mechanisms of tinnitus. Audiology and Neuro-otology. 2002;7(6):358–369. doi: 10.1159/000066156 12401967

[24] Brand T, Hohmann V. An adaptive procedure for categorical loudness scaling. Journal of the Acoustical Society of America. 2002;112(4):1597–1604. doi: 10.1121/1.1502902 12398465

[25] Sanchez Lopez R, Fereczkowski M, Bianchi F, El-Haj-Ali M, Neher T, Dau T, et al. Auditory tests for characterizing individual hearing deficits: The BEAR test battery. 2018;.

[26] Mepani AM, Kirk SA, Hancock KE, Bennett K, De Gruttola V, Liberman MC, et al. Middle Ear Muscle Reflex and Word Recognition in “normal-Hearing” Adults: Evidence for Cochlear Synaptopathy? Ear and Hearing. 2018;41(1):25–38. doi: 10.1097/AUD.0000000000000804 PMC6934902 31584501

[27] Keefe DH, Fitzpatrick D, Liu YW, Sanford CA, Gorga MP. Wideband acoustic-reflex test in a test battery to predict middle-ear dysfunction. Hearing Research. 2010;263(1-2):52–65. doi: 10.1016/j.heares.2009.09.008 19772907 PMC3694582

[28] Schlittenlacher J, Turner RE, Moore BCJ. Audiogram estimation using Bayesian active learning. The Journal of the Acoustical Society of America. 2018;144(1):421–430. doi: 10.1121/1.5047436 30075695

[29] R Core Team. R: A Language and Environment for Statistical Computing; 2020. Available from: https://www.R-project.org/.

[30] Bezdek JC. Pattern recognition with fuzzy objective function algorithms. Plenum; 1981.

[31] Ramdani MA, Abdullah S. Application of partitioning around medoids cluster for analysis of stunting in 100 priority regencies in Indonesia. Journal of Physics: Conference Series. 2021;1722(1):012097.

[32] Archer KJ, Kimes RV. Empirical characterization of random forest variable importance measures. Computational Statistics and Data Analysis. 2008;52(4):2249–2260. doi: 10.1016/j.csda.2007.08.015

[33] Sutton CD. Classification and Regression Trees, Bagging, and Boosting. Handbook of Statistics. 2005;24:303–329. doi: 10.1016/S0169-7161(04)24011-1

[34] Frazier PI. A Tutorial on Bayesian Optimization. 2018;.

[35] Cicchetti DV. Guidelines, criteria, and rules of thumb for evaluating normed and standardized assessment instruments in psychology. Psychological Assessment. 1994;6(4):284–290. doi: 10.1037/1040-3590.6.4.284

[36] Koo TK, Li MY. A Guideline of Selecting and Reporting Intraclass Correlation Coefficients for Reliability Research. Journal of Chiropractic Medicine. 2016;15(2):155–163. doi: 10.1016/j.jcm.2016.02.012 27330520 PMC4913118

[37] Kodama A, Kitahara M. Clinical and audiological characteristics of tonal and noise tinnitus. Orl-journal for Oto-rhino-laryngology and Its Related Specialties. 1990;52(3):156–163. doi: 10.1159/000276126 2359592

[38] Valero MD, Hancock KE, Maison SF, Liberman MC. Effects of cochlear synaptopathy on middle-ear muscle reflexes in unanesthetized mice. Hearing Research. 2018;363:109–118. doi: 10.1016/j.heares.2018.03.012 29598837 PMC6359722

[39] Han TS, Jeong JE, Park SN, Kim JJ. Gender differences affecting psychiatric distress and tinnitus severity. Clinical Psychopharmacology and Neuroscience. 2019;17(1):113–120. doi: 10.9758/cpn.2019.17.1.113 30690946 PMC6361036

[40] Fioretti A, Natalini E, Riedl D, Moschen R, Eibenstein A. Gender Comparison of Psychological Comorbidities in Tinnitus Patients—Results of a Cross-Sectional Study. Frontiers in Neuroscience. 2020;14(July):1–11. doi: 10.3389/fnins.2020.00704 32774239 PMC7381348

